# Clinical standards for drug-susceptible pulmonary TB

**DOI:** 10.5588/ijtld.22.0228

**Published:** 2022-07-01

**Authors:** O. W. Akkerman, R. Duarte, S. Tiberi, H. S. Schaaf, C. Lange, J. W. C. Alffenaar, J. Denholm, A. C. C. Carvalho, M. S. Bolhuis, S. Borisov, J. Bruchfeld, A. M. Cabibbe, J. A. Caminero, I. Carvalho, J. Chakaya, R. Centis, M. P. Dalcomo, L. D’Ambrosio, M. Dedicoat, K. Dheda, K. E. Dooley, J. Furin, J-M. García-García, N. A. H. van Hest, B. C. de Jong, X. Kurhasani, A. G. Märtson, S. Mpagama, M. Munoz. Torrico, E. Nunes, C. W. M. Ong, D. J. Palmero, R. Ruslami, A. M. I. Saktiawati, C. Semuto, D. R. Silva, R. Singla, I. Solovic, S. Srivastava, J. E. M. de Steenwinkel, A. Story, M. G. G. Sturkenboom, M. Tadolini, Z. F. Udwadia, A. R. Verhage, J. P. Zellweger, G. B. Migliori

**Affiliations:** 1 TB Center Beatrixoord, University Medical Center Groningen, University of Groningen, Haren; 2 Department of Pulmonary Diseases and Tuberculosis, University Medical Center Groningen, University of Groningen, Groningen, the Netherlands; 3 Centro Hospitalar de Vila Nova de Gaia/Espinho; Instituto de Ciencias Biomédicas de Abel Saalazar, Universidade do Porto, Instituto de Saúde Publica da Universidade do Porto, Unidade de Investigação Clínica, ARS Norte, Porto, Portugal; 4 Blizard Institute, Barts and The London School of Medicine and Dentistry, Queen Mary University of London, Division of Infection, Royal London Hospital, Barts Health NHS Trust, London, UK; 5 Desmond Tutu TB Centre, Department of Paediatrics and Child Health, Faculty of Medicine and Health Sciences, Stellenbosch University, Cape Town, South Africa; 6 Division of Clinical Infectious Diseases, Research Center Borstel, Borstel, Germany; 7 German Center for Infection Research (DZIF) Clinical Tuberculosis Unit, Borstel, Germany; 8 Respiratory Medicine & International Health, University of Lübeck, Lübeck, Germany; 9 The Global Tuberculosis Program, Texas Children’s Hospital, Immigrant and Global Health, Department of Pediatrics, Baylor College of Medicine, Houston, TX, USA; 10 Sydney Institute for Infectious Diseases, The University of Sydney, Sydney, NSW, Australia; 11 School of Pharmacy, The University of Sydney Faculty of Medicine and Health, Sydney, NSW, Australia; 12 Westmead Hospital, Sydney, NSW, Australia; 13 Victorian Tuberculosis Program, Melbourne Health, Department of Infectious diseases, University of Melbourne, at the Peter Doherty Institute for Infection and Immunity, Melbourne, VIC, Australia; 14 Laboratório de Inovaçôes em Terapias, Ensino e Bioprodutos, Instituto Oswaldo Cruz, Fundação Oswaldo Cruz (FIOCRUZ), Rio de Janeiro, RJ, Brazil; 15 Department of Clinical Pharmacy and Pharmacology, University of Groningen, University Medical Center Groningen, Groningen, the Netherlands; 16 Moscow Research and Clinical Center for Tuberculosis Control, Moscow, Russia; 17 Division of Infectious Diseases, Department of Medicine, Karolinska Institutet, Solna, Stockholm, Sweden; 18 Department of Infectious Disease, Karolinska University Hospital, Stockholm, Sweden; 19 Emerging Bacterial Pathogens Unit, Division of Immunology, Transplantation and Infectious Diseases, Istituto di Ricovero e Cura a Carattere Scientifico (IRCCS) San Raffaele Scientific Institute, Milan, Italy; 20 Department of Pneumology, University General Hospital of Gran Canaria “Dr Negrin”, Las Palmas, Spain; 21 ALOSA (Active Learning over Sanitary Aspects) TB Academy, Spain; 22 Pediatric Department, Vila Nova de Gaia Outpatient Tuberculosis Centre, Vila Nova de Gaia Hospital Centre, Vila Nova de Gaia, Portugal; 23 Department of Medicine, Therapeutics and Dermatology, Kenyatta University, Nairobi, Kenya; 24 Department of Clinical Sciences. Liverpool School of Tropical Medicine, Liverpool, UK; 25 Servizio di Epidemiologia Clinica delle Malattie Respiratorie, Istituti Clinici Scientifici Maugeri IRCCS, Tradate, Italy; 26 Reference Center Helio Fraga, FIOCRUZ, Brazil; 27 Public Health Consulting Group, Lugano, Switzerland; 28 Department of Infectious Diseases, Heartlands Hospital, University Hospitals Birmingham NHS Foundation Trust, Birmingham, UK; 29 Centre for Lung Infection and Immunity Unit, Department of Medicine, Division of Pulmonology and UCT Lung Institute, University of Cape Town, Cape Town, South Africa; 30 South African Medical Research Council Centre for the Study of Antimicrobial Resistance, University of Cape Town, Cape Town, South Africa; 31 Faculty of Infectious and Tropical Diseases, London School of Hygiene & Tropical Medicine, London, UK; 32 Center for Tuberculosis Research, Johns Hopkins, Baltimore, MD; 33 Department of Global Health and Social Medicine, Harvard Medical School, Boston, MA, USA; 34 Tuberculosis Research Programme SEPAR, E-08029 Barcelona, Spain; 35 Municipal Public Health Service Groningen, Groningen, The Netherlands; 36 Mycobacteriology Unit, Department of Biomedical Sciences, Institute of Tropical Medicine, Antwerp, Belgium; 37 UBT-Higher Education Institution Prishtina, Kosovo; 38 Antimicrobial Pharmacodynamics and Therapeutics, Department of Pharmacology and Therapeutics, University of Liverpool, Liverpool, UK; 39 Kilimanjaro Christian Medical University College, Moshi, United Republic of Tanzania; 40 Kibong’oto Infectious Diseases Hospital, Sanya Juu, Siha, Kilimanjaro, United Republic of Tanzania; 41 Clínica de Tuberculosis, Instituto Nacional de Enfermedades Respiratorias Ismael Cosio Villegas, México City, Mexico; 42 Department of Pulmonology of Central Hospital of Maputo, Maputo, Mozambique; 43 Faculty of Medicine of Eduardo Mondlane University, Maputo, Mozambique; 44 Infectious Disease Translational Research Programme, Department of Medicine, National University of Singapore, Yong Loo Lin School of Medicine, Singapore; 45 National University of Singapore Institute for Health Innovation & Technology (iHealthtech), Singapore; 46 Division of Infectious Diseases, Department of Medicine, National University Hospital, Singapore; 47 Faculdade de Medicina, Universidade Federal do Rio Grande do Sul, Porto Alegre, RS, Brazil; 48 Department of Biomedical Science, Faculty of Medicine, Universitas Padjadjaran, Bandung, Indonesia; 49 Research Center for Care and Control of Infectious Disease (RC3iD), Universitas Padjadjaran, Bandung, Indonesia; 50 Department of Internal Medicine, Public Health and Nursing, Universitas Gadjah Mada, Yogyakarta, Indonesia; 51 Center for Tropical Medicine, Faculty of Medicine, Public Health and Nursing, Universitas Gadjah Mada, Yogyakarta, Indonesia; 52 Research, Innovation and Data Science Division, Rwanda Biomedical Center, Kigali, Rwanda; 53 Instituto Vaccarezza, Hospital Muñiz, Buenos Aires, Argentina; 54 National Institute of Tuberculosis & Respiratory Diseases, New Delhi, India; 55 National Institute of Tuberculosis, Lung Diseases and Thoracic Surgery, Faculty of Health, Catholic University, RuŽomberok, Vyšné Hágy, Slovakia; 56 Department of Pulmonary Immunology, University of Texas Health Science Centre at Tyler, Tyler, TX, USA; 57 Department of Medical Microbiology and Infectious Diseases, Erasmus University Medical Centre Rotterdam, Rotterdam, the Netherlands; 58 Institute of Epidemiology and Healthcare, University College London, London, UK; 59 Find and Treat, University College Hospitals NHS Foundation Trust, London, UK; 60 Infectious Diseases Unit, IRCCS Azienda Ospedaliero-Universitaria di Bologna, Bologna, Italy; 61 Department of Medical and Surgical Sciences, Alma Mater Studiorum University of Bologna, Bologna, Italy; 62 P. D. Hinduja National Hospital and Medical Research Centre, Mumbai, India; 63 Department of Pediatrics, University Medical Center Groningen, University of Groningen, Groningen, the Netherlands; 64 TB Competence Center, Swiss Lung Association, Berne, Switzerland

**Keywords:** pulmonary TB, management, diagnosis, treatment, education, rehabilitation, clinical standards

## Abstract

**BACKGROUND:**

The aim of these clinical standards is to provide guidance on ‘best practice’ for diagnosis, treatment and management of drug-susceptible pulmonary TB (PTB).

**METHODS:**

A panel of 54 global experts in the field of TB care, public health, microbiology, and pharmacology were identified; 46 participated in a Delphi process. A 5-point Likert scale was used to score draft standards. The final document represents the broad consensus and was approved by all 46 participants.

**RESULTS:**

Seven clinical standards were defined: Standard 1, all patients (adult or child) who have symptoms and signs compatible with PTB should undergo investigations to reach a diagnosis; Standard 2, adequate bacteriological tests should be conducted to exclude drug-resistant TB; Standard 3, an appropriate regimen recommended by WHO and national guidelines for the treatment of PTB should be identified; Standard 4, health education and counselling should be provided for each patient starting treatment; Standard 5, treatment monitoring should be conducted to assess adherence, follow patient progress, identify and manage adverse events, and detect development of resistance; Standard 6, a recommended series of patient examinations should be performed at the end of treatment; Standard 7, necessary public health actions should be conducted for each patient. We also identified priorities for future research into PTB.

**CONCLUSION:**

These consensus-based clinical standards will help to improve patient care by guiding clinicians and programme managers in planning and implementation of locally appropriate measures for optimal person-centred treatment for PTB.

## Abstract (fr)

**CONTEXTE:**

L’objectif de ces normes cliniques est de fournir des conseils sur les “meilleures pratiques” en matière de diagnostic, de traitement et de prise en charge de la tuberculose pulmonaire (PTB) pharmacosensible.

**MÉTHODES:**

Un panel de 54 experts mondiaux dans le domaine des soins antituberculeux, de la santé publique, de la microbiologie et de la pharmacologie a été identifié ; 46 ont participé à un processus Delphi. Une échelle de Likert en 5 points a été utilisée pour noter les projets de normes. Le document final représente le large consensus et a été approuvé par les 46 participants.

**RÉSULTATS:**

Sept normes cliniques ont été définies : Norme 1, tous les patients (adultes ou enfants) qui présentent des symptômes et des signes compatibles avec une PTB doivent subir des examens pour parvenir à un diagnostic ; Norme 2, des tests bactériologiques adéquats doivent être effectués pour exclure une TB résistante aux médicaments ; Norme 3, un régime convenable recommandé par l’OMS et les directives nationales pour le traitement de la PTB doit être identifié ; Norme 4, une éducation et des conseils sur la santé doivent être dispensés à chaque patient commençant le traitement ; Norme 5, un suivi du traitement doit être effectué pour évaluer l’adhésion, suivre les progrès du patient, identifier et gérer les effets indésirables et détecter le développement de la résistance ; Norme 6, une série recommandée d’examens du patient doit être effectuée à la fin du traitement ; Norme 7, les actions de santé publique nécessaires doivent être menées pour chaque patient. Nous avons également identifié les priorités pour les recherches futures sur la PTB.

**CONCLUSION:**

Ces normes cliniques consensuelles contribueront à améliorer la prise en charge des patients en guidant les cliniciens et les responsables de programmes dans la planification et la mise en œuvre de mesures localement appropriées pour un traitement optimal de la PTB centré sur la personne.

According to WHO estimates, there were 9.9 million TB cases in 2020, with 1.5 million deaths (including those co-infected with HIV).^1^ These numbers may be an underestimate as the COVID-19 pandemic set back global TB control services, leading to underdiagnosed TB disease and increased spread of TB. In 2020, the gap between diagnosed and reported patients vs. estimated patients was 4.1 million cases.^1^ This implies that further and larger steps are needed to reach the targets of reducing TB incidence and deaths set by the WHO End TB Strategy.^2^

Access to rapid diagnosis with drug susceptibility testing (DST) results and effective treatment is essential to control TB. However, this is not possible unless symptoms and signs indicating pulmonary TB (PTB) are correctly identified based on knowledge of the most common risk factors for TB disease, followed by appropriate diagnostics. If PTB is microbiologically confirmed, rapid molecular DST is essential for selecting an appropriate treatment regimen. An effective regimen requires the use of effective drugs at correct dosages for the appropriate duration of treatment. To prevent the selection for drug-resistant mycobacteria, optimal treatment of drug-susceptible TB (DS-TB) requires three or more effective drugs in the intensive phase and two or more effective drugs in the continuation phase, along with adequate exposure to these drugs.^3^ Substantial variations in exposure to both isoniazid (INH, H) and rifampicin (RIF, R) have been shown in pharmacokinetic studies.^4^–^8^ Inadequate exposure can be caused by malabsorption (malnutrition, severe state of disease, gastro-intestinal tract problems and HIV coinfection), delayed absorption due to diabetes mellitus (DM), increased clearance rate (smoking) and drug-drug interactions with INH, fast *N*-acetyl-transferase-2 acetylator status.^9^–^11^

Treatment adherence is a well-known challenge for a successful outcome. It is important to know why some patients discontinue treatment or take irregular treatment.^12^ Adherence is influenced by factors such as knowledge of the disease, the reason for treatment, treatment duration, adverse events during treatment and social determinants (such as costs of TB treatment, loss of income during treatment, hospital stay, isolation, substance abuse and stigma).^13^ The current standard DS-TB regimen has been used for over 40 years. In 2021, a 4-month regimen comprising INH, pyrazinamide (PZA, Z), rifapentine (P) and moxifloxacin (Mfx) was shown to be non-inferior to the standard 6-month DS-TB regimen.^14^ This shorter regimen could potentially signify a paradigm shift in the management of DS-TB treatment.

The IJTLD Clinical Standards for Lung Health complement existing WHO or other guidelines and integrate their recommendations to provide a specific clinical focus.^8^,^15^–^19^ The standards are universal principles and might need to be adapted to specific settings and situations for future programmatic implementation for legal, organisational or economic reasons. Specific evidence in some areas is still limited. The clinical standards presented here are based on the best available evidence and will be updated in due course to capture new evidence as it accumulates. The clinical standards are not intended to create discrimination. Differences in capacity and access to technology between high- and low-resource healthcare facilities mean that some settings or services may not be able to meet all standards. Nevertheless, these standards can contribute to ensuring the highest standard of care for all patients with PTB.

## AIM OF THE CLINICAL STANDARDS

This consensus-based document describes the following standards:

1. Every individual (child and adult) with symptoms and signs compatible with PTB should undergo the necessary diagnostic investigations (Standard 1).
2. All patients with symptoms and signs compatible with PTB should undergo bacteriological tests (Standard 2).
3. All patients with PTB should be treated with an appropriate regimen as recommended by the WHO and/or national guidelines (Standard 3).
4. All patients initiating treatment for PTB should be provided health education/counselling (Standard 4).
5. Treatment monitoring should be conducted to follow the patient’s progress, support patients during treatment, assess treatment adherence, detect and manage adverse effects early and detect the emergence of resistance to anti-TB drugs (Standard 5).
6. At the end of treatment for PTB, a set of checks should be performed for each patient (Standard 6).
7. For each patient with PTB, a set of public health actions should be conducted (Standard 7).

In addition, future research priorities for PTB are highlighted.

## METHODS

A panel of global experts was identified to represent the main scientific societies, associations and groups active in TB. Of the 54 experts initially invited, six did not respond after one reminder. The remaining 48 respondents were asked to comment using a Delphi process on an initial draft of seven Standards developed by a core coordination team composed of six members (OA, GBM, RD, RC, LA, ST). Of these, 46 provided valid answers. The final panel included TB clinicians (*n* = 30), TB public health specialists (*n =* 4), TB paediatricians (*n* = 3), pharmacologists (*n* = 5) and microbiologists/biologists (*n* = 4). A 5-point Likert scale was used (5: high agreement; 1: low agreement). At the first Delphi round, the agreement was high with a median value of >4.4 (for all standards), and no major changes were made to the draft standards. Based on this substantial agreement, the expert panel jointly developed a draft document. The document underwent seven rounds of revision, and the final version was approved by consensus (100% agreement).

## STANDARD 1

**Every individual (child or adult) with symptoms and signs compatible with PTB should be examined and undergo the recommended investigations.**

All individuals with presumed PTB should be examined and undergo investigations as part of the diagnostic process, based and organised according to the availability of services and resources so that treatment can be started. The complete list of recommended investigations to assess the presence of PTB are given in Table 1. A focused history needs to be recorded for all patients, including history of TB contact, as well as the DST of the source case if available, previous TB disease, previous TB treatment or preventive therapy, symptoms and signs of PTB, history of comorbidities, smoking, illicit drug and/or alcohol abuse, incarceration, migration from (or travel history in) a country with high incidence of TB and/or HIV, and treatment with biologicals or other immunosuppressive drugs. Known symptoms and signs highly associated with PTB are cough, haemoptysis, fever, night sweats, fatigue, loss of appetite and weight loss; in children, failure to thrive and decreased playfulness are also symptoms. Except for weight loss or failure to thrive, the duration of these symptoms and signs is less important. Cases will be missed if cut-off values for duration are established. A study and survey in South Africa showed that 60% of TB patients had complaints of cough of <2 weeks.^20^ Percentage and duration of weight loss are less important for PTB diagnosis but essential for nutritional assessment. Further history and examination should include assessing comorbid medical conditions associated with unfavourable treatment outcomes and increased morbidity and mortality in TB patients. These include malnutrition, HIV infection, DM, chronic kidney diseases, liver disease (hepatitis B and C), alcohol abuse and social deprivation.^21^–^27^ Globally, malnutrition and HIV infection (especially, if not on antiretroviral therapy [ART]) are the main risk factors for the reactivation of TB.^28^–^30^ Furthermore, this leads to muscle depletion (sarcopenia), decreased mental well-being and ultimately, slower reintegration into societal participation of patients during treatment.^21^ Alcohol use, smoking cigarettes, illicit drug and/or opiate substitute use are further risk factors for TB reactivation, but can also complicate treatment.^31^–^36^ For opiate replacement therapy, an additional challenge of drug-drug interaction with RIF may result in lower exposure to methadone, leading to withdrawal symptoms and complicating treatment adherence.^37^

**Table 1:** Investigations, recommended and possible investigations

Investigations	Recommended investigations	Possible investigations, when clinically indicated and available	Comments
Symptoms and signs	Cough Haemoptysis Fever Night sweats Fatigue Loss of appetite Weight loss/failure to thrive Reduced playfulness		
Imaging	Chest radiography	Chest radiography (digital) Chest CT scan	Chest CT for complicated intrathoracic TB or if TB considered but chest radiograph was non-specific
Microbiology	Smear microscopy for AFB Molecular testing for *M. tuberculosis* and DST Mycobacterial culture and DST (if available)	Whole-genome sequencing	Mainly sputum, but any possible respiratory specimen in children (e.g., gastric aspirate, induced sputum, stool)
Assessment of comorbidities	Malnutrition, HIV, diabetes mellitus	Chronic kidney disease, liver disease	
Assessment of substance abuse	Cigarette smoking, alcohol use, illicit drug and/or methadone use		

CT =computed tomography; AFB = acid-fast bacilli; DST = drug susceptibility testing

Chest radiography (CXR) is important for PTB diagnosis, especially in immunocompetent patients, and gives information on the extent of pulmonary disease, including consolidation and/or cavities.^38^ A chest computed tomography (CT) scan is more sensitive for smaller intrapulmonary lesions and for atypical presentations in immunocompromised patients. CXR is the most common diagnostic tool for TB in children, and often supports the diagnosis of intrathoracic TB, including mediastinal lymphadenopathy and pleural effusions. Although cavities are rare in children, enlargement of perihilar or paratracheal lymph nodes, bronchial compression/deviation, miliary infiltrates and pleural effusions are quite specific for paediatric intrathoracic TB.^39^,^40^

Microbiological confirmation of PTB should be determined by examining respiratory samples (e.g., sputum, gastric aspirate/lavage or bronchoalveolar lavage). Instructions on sputum collection should be provided to adults and older children when securing an early morning sputum sample, plus a second spot sputum or two spot sputum samples separated in time.^41^ The samples should be collected, preferably within 24 h, and be processed within hours to assure rapid diagnostic confirmation. If the initial sputum samples are negative, sputum induction can be performed to obtain better specimens.^42^,^43^ Obtaining respiratory specimens from (younger) children is challenging, but children aged >6 years should be able to expectorate sputum. If not successful, every effort should still be made to obtain alternative respiratory specimens for microbiological examination, such as gastric aspirates/lavage, induced sputum, nasopharyngeal aspirate and/or stool (stool for Xpert^®^ MTB/RIF [Ultra] only; Cepheid, Sunnyvale, CA, USA). If intubated or if bronchoscopy is done, tracheal aspirates or bronchoalveolar lavage, respectively, should be obtained to obtain samples for microbiology for confirmation and to exclude other pathologies (including cancer).

## STANDARD 2

**All patients with symptoms and signs compatible with PTB should undergo a set of bacteriological tests.**

Following the examinations done under Standard 1, adequate bacteriological analysis should be conducted in a timely manner to both confirm cases and perform DST. All patients should undergo molecular *Mycobacterium tuberculosis* complex identification and DST as the initial diagnostic test. All patients should have specimens sent for mycobacterial culture and phenotypic DST, if needed. Drug-resistant TB (DR-TB), including multidrug-resistant TB (MDR-TB) or extensively drug-resistant TB (XDR-TB), needs to be excluded to prevent undertreatment. Solid or liquid culture is the conventional diagnostic tool for growth of acid-fast bacilli (AFB), including *M. tuberculosis* complex, and phenotypic DST. Sputum smear microscopy for AFB remains important to assess the patient’s potential infectious state, which is important for public health actions. Rapid molecular tests for the diagnosis of TB with drug resistance detection, such as Xpert MTB/RIF (Ultra; Cepheid), Truenat MTB (Plus) (Molbio Diagnostics, Verna, India) and moderate-complexity automated nucleic-acid amplification tests (NAATs; Abbott RealTime MTB RIF/INH [Abbott Laboratories, Chicago, IL, USA], BD MAX^™^ MDR-TB [BD, Franklin Lakes, NJ, USA], FluoroType MTBDR [Hain Lifescience, Nehren, Germany] and Roche Cobas MTB RIF/INH assay [Roche, Basel, Switzerland]) have been developed to provide a more timely result both for diagnosis and for drug susceptibility than phenotypic tests.^44^,^45^ All tests can identify mutations associated with RIF resistance (in the *rpo*B gene that confer RIF resistance) and, in the case of Truenat MTB (Plus) and the moderate complexity automated NAATs, can also identify mutations associated with INH (in the *kat*G gene and the *inh*A promotor region), that is, these are able to detect MDR-TB.^46^,^47^ In case of sputum smear-positive specimens or a cultured isolate of *M. tuberculosis* complex, commercial molecular line-probe assays (LPAs), such as the GenoType MTBDR*plus* (Hain Lifescience) and the INNO-LiPA Rif.TB (Innogenetics, Ghent, Belgium), can also be used to detect resistance to RIF and INH. However, some RIF- and INH-conferring mutations may be missed, and these mutations may vary in different settings.^48^ If RIF resistance is detected, the Xpert MTB/XDR can detect resistance to INH, the fluoroquinolones, second-line injectable drugs and ethionamide,^49^–^53^ while the GenoType MTBDR*sl* assay (Hain Life-science) can be used to detect resistance to fluoroquinolones and the second-line injectable drugs.^45^

**Table 2:** Guidance regarding bacteriological investigations

For each patient with symptoms and signs compatible with PTB, a set of bacteriological investigation should be conducted in a timely manner: Specimen collection (preferably within 24 h) Specimen processing within 72 h All patients should have molecular mycobacterial identification and DST as the initial diagnostic test All cultured isolates should be submitted to molecular and/or phenotypical DST If available, cultures should be submitted for DST and whole or next genome sequencing. In case of economic constraints, this should be done at least in case of outbreak or RR/MDR-TB case

DST = drug susceptibility testing; RR/MDR-TB = rifampicin-resistant/multidrug-resistant TB.

Next-generation sequencing (NGS), either by whole-genome sequencing (WGS) or targeted (amplicon) sequencing is an important tool to inform clinical and public health practice.^54^ In some settings, NGS technologies have already become the basis of drug resistance surveillance and outbreak analysis.^55^ However, sequencing technologies require more DNA than amplification technologies (e.g., Xpert), and are therefore usually only reliably performed on cultured specimen. Targeting only genes where mutations are associated with resistance to anti-TB drugs (amplicons) needs less bacterial DNA than WGS and can be used for rapid molecular prediction of resistance against all anti-TB drugs when AFB are visible on sputum smear microscopy.^56^–^58^ When facing an outbreak in the community or primary isolation of a RR/MDR-TB resistant strain of *M. tuberculosis*, the specimen or cultured isolate should be submitted for NGS.^59^–^64^

## STANDARD 3

**All patients with PTB should be treated with an appropriate regimen as recommended by the WHO and/or national guidelines.**

All patients diagnosed with PTB should be offered effective treatment as soon as practically possible to improve prognosis as well as to limit transmission. Patient history, including drug allergies and concomitant medications, is important to avoid significant adverse events and drug-drug interactions. Treatment of TB disease can be broadly split into DS-TB and DR-TB treatment. DR-TB treatment is not part of this standard. Patients who have confirmed DS-TB, can be prescribed the WHO-recommended regimen comprising an initial intensive phase of 2 months of INH, RIF, PZA and ethambutol (EMB, E), followed by a 4-month continuation phase with RIF and INH (2HRZE/4HR).^15^ Following molecular (including WGS) or phenotypic DST, the treatment can be modified according to the final DST results; should a patient’s *M. tuberculosis* isolate be pan-susceptible, EMB may be stopped.^19^ Drug dosing is based on patient age and weight as per WHO/national guidelines recommendations. Daily dosing is recommended throughout the course to prevent acquired resistance. There are several formulations available, including single-drug formulations and fixed-dose combinations (FDC). Some of the latter are child-friendly formulations with dispersible tablets at lower milligram contents. Suspensions, such as RIF suspension, are best avoided as these often provide low RIF exposure.^65^ Oral pyridoxine (vitamin B6) should be co-administered with the TB regimen for the duration of INH for those at risk of developing peripheral neuropathy due to either alcohol dependency, malnutrition, DM, HIV infection, pregnancy, lactation, patients with a history of a seizure disorder or elderly patients.^66^

Despite the long duration of anti-TB treatment and variable responses to treatments, recommendations for treatment durations are currently standardised. Biomarkers to individualise the duration of anti-TB therapies are currently under evaluation.^67^ The current recommendation for the duration of PTB treatment with the standard 2HRZ(E)/4HR regimen is 6 months. Monitoring to evaluate treatment response in patients with PTB is recommended; patients should have a repeat sputum for smear and culture at Month 2.^8^,^17^ Duration of this regimen should be extended if delayed sputum smear and culture conversion occurs (confirmed or presumed DS-TB), and/or in case of extensive cavitary disease with slow clinical and/or radiological improvement, provided that an undetected or newly acquired drug resistance has been excluded. Ideally, 4 months of treatment after culture conversion should be administered.^19^ Culture positivity at Month 2 should result in closer follow up, adherence check, repeat DST, repeat CXR, assistance with smoking cessation, review of illicit drugs and alcohol use, optimisation of concurrent medical conditions (e.g., initiating ART for people living with HIV/AIDS and better glucose control in DM patients) and therapeutic drug monitoring.^8^ Duration of DS-TB treatment may be reduced to 4 months with the regimen from Study 31 (2HPZMfx/2HPMfx),^14^ except in certain circumstances, e.g., complex forms of extrapulmonary TB and HIV-infected persons with CD4 <100 cells/ml.^14^,^68^ In children with non-severe (i.e., intrathoracic TB confined to opacification of <1 lobe with no cavities, no signs of miliary TB, no complex pleural effusion and no clinically significant airway obstruction; or only peripheral lymph node TB), drug-susceptible, smear-negative TB, a shorter 4-month regimen of 2HRZ(E)/2HR has recently been shown to be non-inferior to 2HRZ(E)/4HR, and is now recommended by the WHO in children with non-severe PTB (See also Table 3).^69^,^70^ The availability of palatable paediatric formulations and tolerability of medications may help with adherence to treatment.^71^

**Table 3:** Drug-susceptible PTB treatment regimens

Each patient with diagnosis of drug-susceptible PTB should be treated with a regimen recommended by WHO and national guidelines Adults: 6-month regimen (2HRZE/4HR) 4-month regimen (2HPZMfx/2PMfx) for children aged >12 years and adults Children: 6-month regimen (2HRZ(E)*/4HR), with higher R and H dosing (see WHO-recommended dosages) SHINE regimen (2HRZ(E)*/2HR) for age <16 years with non-severe TB^†^

* E not always included, especially if susceptibility to R and H was confirmed.

† Intrathoracic TB confined to opacification of <1 lobe with no cavities, no signs of military TB, no complex pleural effusion and no clinically significant airway obstruction; or only peripheral lymph node TB, drug-susceptible and smear-negative for acid-fast bacilli.

PTB = pulmonary TB; H = isoniazid; R = rifampicin; Z = pyrazinamide; E = ethambutol; P = rifapentine; Mfx = moxifloxacin; SHINE = Shorter Treatment for Minimal Tuberculosis in Children

A person-centred approach including education and allowing for informed decisions regarding treatment options is recommended.^72^ Moreover, treatment and support should be tailored to the patient’s needs, thus building confidence with the patient and ensuring mutual respect.^72^ Treatment adherence is essential for success and for individual patient support several tools are available to monitor adherence to treatment, including directly observed treatment (DOT) and video-observed treatment (VOT).^73^–^75^

## STANDARD 4

**All patients initiating treatment for PTB should be provided health education/counselling.**

Health education and counselling before starting TB treatment, and during follow-up, should be offered to individuals who undergo treatment for PTB, to caregivers (in the case of children with PTB), and possibly to the patient’s family and/or community. This is especially important for children, as parents and/or other caregivers will be responsible for administering medication, and the absence of a responsible caregiver may lead to long-term hospitalisation.^76^ Health education and counselling initiatives should promote knowledge about TB and the rationale for treatment monitoring in order to reduce stigma and improve patient self-esteem. Health education and counselling should be tailored to the individual’s needs and organised according to feasibility and cost-effectiveness criteria, based on the availability of health services. Health education should be age-specific, gender-sensitive, delivered in the patient’s own language and address potential challenges for the caregiver when administering medication.^77^–^80^ Health education is essential for PTB treatment adherence and for ensuring safety by improving patients’ understanding of risks and benefits and providing clear instructions for reporting adherence challenges and adverse events, thereby motivating treatment completion.^72^ Patients need to know when to contact the healthcare provider in the event of symptoms suggestive of adverse effects. To aid early recognition of drug adverse effects and reporting to healthcare providers, the major adverse effects of the TB drugs should be explained.^81^ Healthcare providers should be easily contactable to enable early reporting of any adverse effects of drugs used for PTB treatment.

TB is heavily impacted by social determinants of health, and it is associated with catastrophic costs for the patients and their families, which may reduce treatment adherence. Therefore, the healthcare team should inform patients about their rights and facilitate access to locally available social protection initiatives (e.g., cash transfer, transport assistance, nutritional support). Furthermore, the counselling and education session may be an opportunity to promote other healthy lifestyle behaviours, including good nutrition or smoking cessation, discuss how to combine treatment intake with daily activities to improve treatment adherence, and to highlight or better manage risk factors predisposing to TB disease (e.g., HIV, DM, illicit drug and alcohol use).^16^

Components of education and counselling include the following:

- Basic principles of TB disease (transmission, epidemiology, clinical aspects, treatment and prevention);
- Stigma reduction and self-esteem promotion;
- Recognition of clinical deterioration and information on actions to take;
- Principles and the importance of treatment (drug intake and adherence);
- Recognition of adverse events of treatment and information on actions to take;
- Recognition of signs and symptoms of post-TB lung disease, which might require further investigation and rehabilitation.^16^
- Importance of adequate nutrition;
- Importance of smoking cessation and alcohol abstinence (if applicable);
- Risk of comorbidities (e.g., HIV infection, hepatitis, DM, illicit drug and alcohol use);
- Rationale for public health actions, including screening of contacts and wider investigations in the event of outbreaks.

Recommendations on how to deliver an effective health education and counselling session are summarised in Table 4.

**Table 4:** Components of the health education and counselling session for PTB patients

Health education and counselling are highly recommended for successful implementation of PTB treatment Health education and counselling are important to empower PTB patients to adhere to the prescribed treatment, to recognise adverse events early and report to health services and to implement infection control measures at home Key points for health education: Structured and comprehensive educational programmes are an integral and essential component of the management of PTB treatment Educational programmes should be age-specific, gender- and culturally sensitive, delivered in the local language and extended to mothers and families/households Education should be delivered by professionals who are competent in the relevant subject areas and trained to deliver educational sessions Educational materials and technological support used to deliver them needs to be evaluated in the setting-specific context Recommended topics for counselling: Basic principles of TB disease (transmission, epidemiology, clinical aspects, treatment and prevention) Stigma reduction and self-esteem promotion Recognising clinical deterioration and what actions to undertake Principles and importance of treatment (adherence) Recognising adverse events of treatment and what actions need to be taken Recognising signs and symptoms at the end of treatment, which might require further investigation and rehabilitation Promote adequate nutrition Importance of smoking cessation (if applicable) Risk of comorbidities (e.g., HIV, diabetes mellitus, illicit drug and alcohol use)

PTB = pulmonary TB.

## STANDARD 5

**Treatment monitoring should be conducted to follow each patient’s progress, support patients during treatment, assess treatment adherence, detect and manage adverse effects early and detect the emergence of resistance to anti-TB drugs.**

Treatment monitoring should be conducted during the whole course of treatment and include the assessment of treatment adherence. As part of a person-centred approach to adherence, an individual strategy should be used.^82^ The DOT strategy has been widely implemented, but adapted strategies such as VOT and digital adherence technologies (DAT) are useful and can improve cost-effectiveness of treatment monitoring as well.^75^,^83^–^88^ During treatment, the assessment of adverse effects is also important, as these may lead to poor adherence and treatment failure.^89^ Knowledge of the adverse effects of each drug is important for both patient and healthcare worker.^81^ Pharmacovigilance is important for new drugs and should be instituted globally for all new drugs.^90^–^92^ Hepatotoxicity is the most common adverse effect, especially for those with comorbidities such as (viral) hepatitis, and liver function tests for monitoring are required. Colour vision should be assessed at baseline and monitored in all patients receiving EMB, especially if for >2 months. Weight should be assessed at each visit. Furthermore, follow-up of sputum smear and culture, where available, is important to monitor treatment success or failure, including in children if PTB was bacteriologically confirmed. If there is no sputum culture conversion after 2 months, DST should be repeated.

A clear relationship between drug exposure, pathogen susceptibility and treatment response has been demonstrated in both DS- and DR-TB.^93^,^94^ With therapeutic drug monitoring (TDM), dosage is adjusted based on drug exposure to ensure therapeutic concentrations improve treatment success and reduce the possibility of toxicity and adverse events.^10^,^95^,^96^ TDM is especially useful in patients at risk of altered drug exposure and severe disease,^3^,^8^,^97^,^98^ and may also be useful in checking adherence. TDM is not currently recommended for all TB patients on treatment, mainly because of availability, time and financial costs, rather than its potential benefit. TDM is at present recommended for certain patient groups: 1) patients who are taking several concomitant medications so as to reduce toxicity, 2) patients with inadequate treatment response (i.e., patients who are not smear microscopy or culture converting and/or have slow clinical and / or radiological improvement), 3) patients with gastrointestinal abnormalities that precipitate malabsorption, 4) those with renal insufficiency, 5) HIV co-infected patients, 6) diabetic patients and 7) those with severe disease, including TB meningitis.^95^,^99^ Innovation in the field of TDM by point-of-care tests using microsampling techniques (such as dried blood spot [DBS] and volumetric absorption microsampling [VAMS]) or non-invasive saliva and urine sampling may help to facilitate implementation in low-resource settings.^100^–^106^

## STANDARD 6

**At the end of treatment for PTB a set of examinations should be performed for each patient.**

At the end of treatment, it is important to record the patient’s clinical history and examine them to determine treatment success and evaluate the presence of post-TB lung disease (which might require rehabilitation), or any adverse effects due to the treatment provided.^16^ Whenever possible, a sputum specimen for smear microscopy and culture should be obtained before completing treatment in patients with bacteriologically confirmed TB who can still produce a voluntary sputum (children often have negative TB bacteriology, so follow-up specimens for bacteriology are not indicated). However, many patients will be unable to produce adequate sputum specimens at this stage, and some national programmes do not recommend a final bacteriological examination when patients show clinical and radiological improvement. A CXR will allow identification of lung sequelae and when severely abnormal, a chest CT scan should be performed (if available and affordable) to better characterise the lung sequelae and allow better evaluation in the future.^16^ If the patient has dyspnoea (following a dyspnoea score) or in the presence of lung sequelae, pulmonary function tests should be provided if available, including a 6-minute walk test, diffusing capacity for carbon oxide (DLCO) and carbon oxide transfer coefficient (KCO) to evaluate post-TB lung disease.^16^ If the patient has low peripheral oxygen saturation, arterial blood gas analysis should also be conducted.

Patients should be followed for at least 6 months after treatment completion, particularly if there is post-TB lung disease, such as bronchiectasis, persisting opacification or lung nodules (and rehabilitation may be necessary).^16^ This time to follow-up can be extended depending on the severity of the sequelae (see also Table 5).

**Table 5:** Set of examinations to be performed at the end of treatment of each patient with PTB

Clinical assessment	Clinical history Symptom assessment Clinical examination
Imaging	Chest radiography Computed tomography if chest radiography is severely abnormal or low dyspnoea score
Microbiological evaluation	If available Sputum specimen for smear microscopy and mycobacterial culture – DST if culture-positive (if possible)
Subjective evaluation	Dyspnoea score
Functional evaluation (if dyspnoea is present)	Six-minute walk test Spirometry Body plethysmography Diffusion capacity assessment (DLCO, KCO) Tidal volume Pulse oximetry Arterial blood gas analysis in case of low peripheral oxygen saturation Cardiopulmonary exercise testing
Plan a follow-up 6 months after TB treatment completion (to evaluate for relapse, bronchiectasis, persisting opacification or nodules which might indicate need for rehabilitation)	

PTB = pulmonary TB; DST = drug susceptibility testing; DLCO = diffusing capacity for carbon monoxide; KCO = carbon monoxide transfer coefficient.

## STANDARD 7

**For each patient with PTB, a set of public health actions should be conducted.**

Because TB is a notifiable infectious disease, there are a series of public health actions to be carried out whenever an individual is diagnosed with the disease. These include timely and complete notification to health authorities (while remaining respectful of people’s right to privacy) and recoding of information in TB registers. This work is important to support TB surveillance and should be done according to international guidelines and national statutory legislation. The TB register (which should be stored in a locked and secured area, or password protected file), should contain information on bacteriological confirmation, including the DST results and sputum smear status, as the latter indicates the patient’s potential infectiousness and consequent need to activate contact tracing according to the ‘stone in the pond’ principle.^107^,^108^ The treatment regimen and the date when the treatment started should be recorded. Understanding patient delay (time from symptom onset to first attempt to seek medical care) and service delay (time from first attempt to seek medical care and date of treatment start) is important to inform targeted public health action to reduce barriers and delays to diagnosis through improved awareness, referral pathways and contact investigations. This is of particular importance for vulnerable household contacts such as children under the age of 5. Ideally, co-existing comorbidities should also be recorded because of their role in the potential treatment outcome, and to improve TB awareness and communication across different clinical specialities.

The WHO has introduced definitions of outcomes, which have been recently revised.^16^,^109^,^110^ These definitions are used by TB programmes for monitoring and evaluation purposes, e.g., to allow them to rapidly calculate the proportion of patients who achieve treatment success (cure, if evidence of bacteriological negativity in a previously positive patient exists, otherwise treatment completion) against those with negative outcomes (e.g., treatment failure, loss to follow-up or death).^109^,^111^–^113^ When revising the definition of cure, the WHO recommends that, when possible and for research purposes, to continue the follow-up of patients for a period of 6 months or 1 year based on the evidence that relapses or re-infections can occur,^109^ thus introducing the concept of ‘sustained cure’. For example, patients undergoing pulmonary rehabilitation offer the possibility of a post-treatment follow-up, as they remain in care and are therefore accessible after completing their anti-TB treatment.

Standard 7 calls for the need to update the TB register if any change occurs in the final outcome (cure or treatment completion), e.g., if the patient relapses (whether recurrence or re-infection) or if death occurs. Communication between the clinical staff and the TB register is encouraged. An additional element of Standard 7 is represented by the importance of prioritising patients with severe post-TB lung disease and disability for access to social protection schemes. This can be based on existing national legislation or facilitated through advocacy to reform or revise legislation in line with Pillar 2 of the WHO End TB Strategy.^16^,^114^,^115^

Finally, in the case of patients moving to different countries, effective trans-border communication is encouraged to provide timely exchange of clinical documents necessary for case referral and ensure optimal continuum of care.^116^ For patients with significant clinical and social challenges to TB management, international platforms providing clinical support are available to complement national TB Consilia, or to provide a second opinion.^97^,^117^–^119^

## PRIORITIES FOR FUTURE RESEARCH INTO TB

There are multiple research priorities for PTB. First, research regarding treatment of PTB should focus on scaling up trial capacity for the testing of new drugs and/or new regimens to identify improved drug combinations for the shortest possible treatment duration. Also, current drugs, such as RIF at a higher dose, should be further investigated. Shorter treatment regimens, preferably with a lower pill burden, which are better tolerated, less toxic and require less monitoring, and which are safe in children, during pregnancy and for people living with HIV, should be developed. Several experts propose a universal drug regimen for both DS- and DR-TB, which may facilitate programmatic implementation; however, growing drug resistance may hamper its effectiveness in the medium term. Treatment research should also focus on better ways to support and enable patients to safely complete treatment as prescribed. This includes differentiated models of care or tailor-made approaches. Second, research into biomarkers that track response to treatment and/or test whether a patient is cured could help clinicians detect failure earlier or reduce duration of treatment. Third, research into reducing implementation gaps and improving patient management algorithms is also needed. Further research priorities include a randomised controlled trial to evaluate TDM, use of inhaled antimicrobial treatment for PTB, and host-directed therapies. Research to better understand post-TB lung disease and mitigation strategies is also urgently needed.

## CONCLUSION

For any person with signs and symptoms of PTB, adequate and timely clinical, radiological and micro-biological assessment is necessary. TB treatment should be based on international guidelines and/or national TB programmes, but certain patients need a more person-centred approach, with consideration of the risks of sub-therapeutic or toxic drug exposure and DST results. Health education and counselling at the start and during treatment can improve treatment adherence and outcomes. Strengthening public health action, most commonly initiated by treating clinicians, is essential to improve TB control and drive elimination. The standards presented here aim to guide best practice and ensure that PTB is diagnosed as early as possible and treated in the best way possible.

## References

[1] World Health Organization (2021). Global tuberculosis report, 2021.

[2] World Health Organization (2014). The End TB Strategy. Global strategy and targets for tuberculosis prevention, care and control after 2015.

[3] Nahid P (2016). Official American Thoracic Society/Centers for Disease Control and Prevention/Infectious Diseases Society of America clinical practice guidelines: Treatment of drug-susceptible tuberculosis. Clin Infect Dis.

[4] Trentalange A (2021). Rifampicin and isoniazid maximal concentrations are below efficacy-associated thresholds in the majority of patients: time to increase the doses. Int J Antimicrob Agents.

[5] Muliaditan M, Della Pasqua O (2019). How long will treatment guidelines for TB continue to overlook variability in drug exposure. J Antimicrob Chemother.

[6] Denti P (2015). Pharmacokinetics of isoniazid, pyrazinamide, and ethambutol in newly diagnosed pulmonary TB patients in Tanzania. PLoS One.

[7] McIlleron H (2006). Determinants of rifampin, isoniazid, pyrazinamide, and ethambutol pharmacokinetics in a cohort of tuberculosis patients. Antimicrob Agents Chemother.

[8] Alffenaar JC (2022). Clinical standards for the dosing and management of TB drugs. Int J Tuberc Lung Dis.

[9] Vogensen VB (2022). Clinical relevance of rifampicin-moxifloxacin interaction in isoniazid-resistant/intolerant tuberculosis patients. Antimicrob Agents Chemother.

[10] Alffenaar JC (2020). Precision and personalized medicine and anti-TB treatment: Is TDM feasible for programmatic use. Int J Infect Dis.

[11] van der Burgt EP (2016). End TB with precision treatment!. Eur Respir J.

[12] Akkerman OW, Tiberi S (2020). The importance of knowing why TB patients stop anti-TB treatment. Int J Tuberc Lung Dis.

[13] Parwati NM (2021). A health belief model-based motivational interviewing for medication adherence and treatment success in pulmonary tuberculosis patients. Int J Environ Res Public Health.

[14] Dorman SE (2021). Four-month rifapentine regimens with or without moxifloxacin for tuberculosis. N Engl J Med.

[15] World Health Organization (2017). Guidelines for treatment of drug-susceptible tuberculosis and patient care.

[16] Migliori GB (2021). Clinical standards for the assessment, management and rehabilitation of post-TB lung disease. Int J Tuberc Lung Dis.

[17] Dlodlo RA (2019). Management of tuberculosis: a guide to essential practice.

[18] World Health Organization (2022). WHO consolidated guidelines on tuberculosis module 5: management of tuberculosis in children and adolescents.

[19] Nahid P (2016). Executive summary: Official American Thoracic Society/Centers for Disease Control and Prevention/Infectious Diseases Society of America clinical practice guidelines: Treatment of drug-susceptible tuberculosis. Clin Infect Dis.

[20] Walt M van der, Moyo S (2018). The First National TB Prevalence Survey, South Africa 2018. https://www.nicd.ac.za/wp-content/uploads/2021/02/TB-Prevalence-survey-report_A4_SA_TPS-Short_Feb-2021.pdf.

[21] ter Beek L (2021). Malnutrition assessment methods in adult patients with tuberculosis: a systematic review. BMJ Open.

[22] Arriaga MB (2022). The effect of diabetes and prediabetes on antituberculosis treatment outcomes: a multicenter prospective cohort study. J Infect Dis.

[23] Akkerman OW (2020). Rehabilitation, optimized nutritional care, and boosting host internal milieu to improve long-term treatment outcomes in tuberculosis patients. Int J Infect Dis.

[24] Dekkers BGJ (2019). Reduced moxifloxacin exposure in patients with tuberculosis and diabetes. Eur Respir J.

[25] Huangfu P (2019). Point of care HbA(1c) level for diabetes mellitus management and its accuracy among tuberculosis patients: a study in four countries. Int J Tuberc Lung Dis.

[26] van Crevel R (2018). Clinical management of combined tuberculosis and diabetes. Int J Tuberc Lung Dis.

[27] Duarte R (2018). Tuberculosis, social determinants and comorbidities (including HIV). Pulmonology.

[28] Hayward S (2018). Factors influencing the higher incidence of tuberculosis among migrants and ethnic minorities in the UK. F1000Res.

[29] Chisti MJ (2013). Pulmonary tuberculosis in severely-malnourished or HIV-infected children with pneumonia: a review. J Health Popul Nutr.

[30] Semba RD (2010). Addressing tuberculosis in the context of malnutrition and HIV coinfection. Food Nutr Bull.

[31] Imtiaz S (2017). Alcohol consumption as a risk factor for tuberculosis: Meta-analyses and burden of disease. Eur Respir J.

[32] Thomas BE (2019). Smoking, alcohol use disorder and tuberculosis treatment outcomes: a dual co-morbidity burden that cannot be ignored. PLoS One.

[33] Ragan EJ (2020). The impact of alcohol use on tuberculosis treatment outcomes: a systematic review and meta-analysis. Int J Tuberc Lung Dis.

[34] Chenciner L (2021). Social and health factors associated with unfavourable treatment outcome in adolescents and young adults with tuberculosis in Brazil: a national retrospective cohort study. Lancet Glob Health.

[35] Louwagie G (2022). Effect of a brief motivational interview and text message intervention targeting tobacco smoking, alcohol use and medication adherence to improve tuberculosis treatment outcomes in adult patients with tuberculosis: a multicentre, randomised controlled trial of the ProLife programme in South Africa. BMJ Open.

[36] Story A, Bothamley G, Hayward A (2008). Crack cocaine and infectious tuberculosis. Emerg Infect Dis.

[37] Kinney EM (2021). Clinical outcomes of concomitant rifamycin and opioid therapy: a systematic review. Pharmacotherapy.

[38] World Health Organization (2016). Chest radiography in tuberculosis detection: summary of current WHO recommendations and guidance on programmatic approaches.

[39] Palmer M (2022). The diagnostic accuracy of chest radiographic features for pediatric intrathoracic tuberculosis. Clin Infect Dis.

[40] Van Hest R (2004). Cavitating tuberculosis in an infant: case report and literature review. Pediatr Infect Dis J.

[41] Datta S (2017). Comparison of sputum collection methods for tuberculosis diagnosis: a systematic review and pairwise and network meta-analysis. Lancet Glob Health.

[42] Luo W (2020). Comparison of sputum induction and bronchoscopy in diagnosis of sputum smear-negative pulmonary tuberculosis: a systemic review and meta-analysis. BMC Pulm Med.

[43] Jiang Q (2019). A randomised controlled trial of stepwise sputum collection to increase yields of confirmed tuberculosis. Int J Tuberc Lung Dis.

[44] Hong JM (2022). Point-of-care diagnostic tests for tuberculosis disease. Sci Transl Med.

[45] World Health Organization (2021). WHO consolidated guidelines on tuberculosis. Module 3: Diagnosis. Rapid diagnostics for tuberculosis detection.

[46] Nathavitharana RR (2017). Accuracy of line probe assays for the diagnosis of pulmonary and multidrug-resistant tuberculosis: a systematic review and meta-analysis. Eur Respir J.

[47] World Health Organization (2016). The use of molecular line probe assays for the detection of resistance to second-line anti-tuberculosis drugs: policy guidance.

[48] Ardizzoni E (2021). Thin-layer-agar-based direct phenotypic drug susceptibility testing on sputum in Eswatini rapidly detects *Mycobacterium tuberculosis* growth and rifampicin resistance otherwise missed by WHO-endorsed diagnostic tests. Antimicrob Agents Chemother.

[49] Naidoo K, Dookie N (2022). Can the GeneXpert MTB/XDR deliver on the promise of expanded, near-patient tuberculosis drug-susceptibility testing. Lancet Infect Dis.

[50] Penn-Nicholson A (2022). Detection of isoniazid, fluoroquinolone, ethionamide, amikacin, kanamycin, and capreomycin resistance by the Xpert MTB/XDR assay: a cross-sectional multicentre diagnostic accuracy study. Lancet Infect Dis.

[51] Georghiou SB (2021). Analytical performance of the Xpert MTB/XDR^®^ assay for tuberculosis and expanded resistance detection. Diagn Microbiol Infect Dis.

[52] Cao Y (2021). Xpert MTB/XDR: a 10-color reflex assay suitable for point-of-care settings to detect isoniazid, fluoroquinolone, and second-line-injectable-drug resistance directly from mycobacterium tuberculosis-positive sputum. J Clin Microbiol.

[53] Bainomugisa A (2020). New Xpert MTB/XDR: added value and future in the field. Eur Respir J.

[54] Dunstan SJ (2019). Understanding the global tuberculosis epidemic: moving towards routine whole-genome sequencing. Int J Tuberc Lung Dis.

[55] Grobbel HP (2021). Design of multidrug-resistant tuberculosis treatment regimens based on DNA sequencing. Clin Infect Dis.

[56] Jouet A (2021). Deep amplicon sequencing for culture-free prediction of susceptibility or resistance to 13 anti-tuberculous drugs. Eur Respir J.

[57] Feuerriegel S (2021). Rapid genomic first- and second-line drug resistance prediction from clinical *Mycobacterium tuberculosis* specimens using Deeplex-MycTB. Eur Respir J.

[58] Lange C (2019). Management of drug-resistant tuberculosis. Lancet.

[59] Hu T (2021). Next-generation sequencing technologies: an overview. Hum Immunol.

[60] Cabibbe AM (2020). Application of targeted next-generation sequencing assay on a portable sequencing platform for culture-free detection of drug-resistant tuberculosis from clinical samples. J Clin Microbiol.

[61] Meehan CJ (2019). Whole genome sequencing of *Mycobacterium tuberculosis* current standards and open issues. Nat Rev Microbiol.

[62] Nimmo C (2019). Whole genome sequencing *Mycobacterium tuberculosis* directly from sputum identifies more genetic diversity than sequencing from culture. BMC Genomics.

[63] McNerney R (2017). Removing the bottleneck in whole genome sequencing of *Mycobacterium tuberculosis* for rapid drug resistance analysis: a call to action. Int J Infect Dis.

[64] Brown AC (2015). Rapid whole-genome sequencing of *Mycobacterium tuberculosis* isolates directly from clinical samples. J Clin Microbiol.

[65] Bekker A (2016). Pharmacokinetics of rifampin, isoniazid, pyrazinamide, and ethambutol in infants dosed according to revised WHO-recommended treatment guidelines. Antimicrob Agents Chemother.

[66] Snider DE (1980). Pyridoxine supplementation during isoniazid therapy. Tubercle.

[67] Heyckendorf J (2021). Prediction of anti-tuberculosis treatment duration based on a 22-gene transcriptomic model. Eur Respir J.

[68] Carr W (2022). Interim guidance: 4-month rifapentine-moxifloxacin regimen for the treatment of drug-susceptible pulmonary tuberculosis - United States, 2022. MMWR Morb Mortal Wkly Rep.

[69] World Health Organization (2021). Rapid communication on updated guidance on the management of tuberculosis in children and adolescents.

[70] Turkova A (2022). Shorter treatment for nonsevere tuberculosis in African and Indian children. N Engl J Med.

[71] Taneja R (2015). Paediatric formulations of second-line anti-tuberculosis medications: challenges and considerations. Int J Tuberc Lung Dis.

[72] Horter S (2021). Person-centred care in TB. Int J Tuberc Lung Dis.

[73] Karumbi J, Garner P (2015). Directly observed therapy for treating tuberculosis. Cochrane Database Syst Rev.

[74] Sinkou H (2017). Video-observed treatment for tuberculosis patients in Belarus: findings from the first programmatic experience. Eur Respir J.

[75] Story A (2019). Smartphone-enabled video-observed versus directly observed treatment for tuberculosis: a multicentre, analyst-blinded, randomised, controlled superiority trial. Lancet.

[76] Musonda HK (2022). Paediatric admissions to a TB hospital: reasons for admission, clinical profile and outcomes. Int J Tuberc Lung Dis.

[77] Fekadu G (2020). Adherence to anti-tuberculosis treatment among pediatric patients at nekemte specialized hospital, Western Ethiopia. Patient Prefer Adherence.

[78] van Zyl S (2006). Adherence to anti-tuberculosis chemoprophylaxis and treatment in children. Int J Tuberc Lung Dis.

[79] Okethwangu D (2019). Multidrug-resistant tuberculosis outbreak associated with poor treatment adherence and delayed treatment: Arua District, Uganda, 2013–2017. BMC Infect Dis.

[80] Cutler RL (2018). Economic impact of medication non-adherence by disease groups: a systematic review. BMJ Open.

[81] Alipanah N (2018). Adherence interventions and outcomes of tuberculosis treatment: A systematic review and meta-analysis of trials and observational studies. PLoS Med.

[82] World Health Organization (2017). Ethical issues in tuberculosis prevention, treatment & care. https://www.whoint/tb/publications/ethics_in_tb_fact-sheet_28jan11revpdf2017.

[83] Ravenscroft L (2020). Video-observed therapy and medication adherence for tuberculosis patients: randomised controlled trial in Moldova. Eur Respir J.

[84] Nsengiyumva NP (2018). Evaluating the potential costs and impact of digital health technologies for tuberculosis treatment support. Eur Respir J.

[85] Acosta J (2022). A randomised controlled trial to evaluate a medication monitoring system for TB treatment. Int J Tuberc Lung Dis.

[86] Getachew E, Woldeamanuel Y, Manyazewal T (2022). Digital health interventions in the clinical care and treatment of tuberculosis and HIV in Central Ethiopia: an initial provider perceptions and acceptability study using the unified theory of acceptance and use of technology model. Int J Mycobacteriol.

[87] McQuaid CF (2021). Digital adherence technology for TB: focus on livelihoods as well as lives. Int J Tuberc Lung Dis.

[88] Zaidi HA, Wells CD (2021). Digital health technologies and adherence to tuberculosis treatment. Bull World Health Organ.

[89] Kim HW (2020). Reasons why patients with tuberculosis in South Korea stop anti-TB treatment: a cross-sectional study. Int J Tuberc Lung Dis.

[90] Akkerman O (2019). Surveillance of adverse events in the treatment of drug-resistant tuberculosis: a global feasibility study. Int J Infect Dis.

[91] Koirala S (2021). Outcome of treatment of MDR-TB or drug-resistant patients treated with bedaquiline and delamanid: results from a large global cohort. Pulmonology.

[92] Borisov S (2019). Surveillance of adverse events in the treatment of drug-resistant tuberculosis: first global report. Eur Respir J.

[93] Zheng X (2021). Drug exposure and minimum inhibitory concentration predict pulmonary tuberculosis treatment response. Clin Infect Dis.

[94] Zheng X (2022). Drug exposure and susceptibility of second-line drugs correlate with treatment response in patients with multidrug-resistant tuberculosis: a multicentre prospective cohort study in china. Eur Respir J.

[95] Märtson AG (2021). Therapeutic drug monitoring in patients with tuberculosis and concurrent medical problems. Expert Opin Drug Metab Toxicol.

[96] Ghimire S (2016). Incorporating therapeutic drug monitoring into the world health organization hierarchy of tuberculosis diagnostics. Eur Respir J.

[97] Nahid P (2019). Treatment of drug-resistant tuberculosis. an official ATS/CDC/ERS/IDSA clinical practice guideline. Am J Respir Crit Care Med.

[98] Alffenaar JC (2020). Integrating pharmacokinetics and pharmacodynamics in operational research to end tuberculosis. Clin Infect Dis.

[99] Galvin J (2022). Pulmonary tuberculosis in intensive care setting, with a focus on the use of severity scores, a multinational collaborative systematic review. Pulmonology.

[100] Alffenaar JC (2021). A mobile microvolume UV/visible light spectrophotometer for the measurement of levofloxacin in saliva. J Antimicrob Chemother.

[101] Mohamed S (2021). Levofloxacin pharmacokinetics in saliva as measured by a mobile microvolume UV spectrophotometer among people treated for rifampicin-resistant TB in Tanzania. J Antimicrob Chemother.

[102] Szipszky C (2021). Determination of rifampin concentrations by urine colorimetry and mobile phone readout for personalized dosing in tuberculosis treatment. J Pediatric Infect Dis Soc.

[103] Hofman S (2015). Role of therapeutic drug monitoring in pulmonary infections: use and potential for expanded use of dried blood spot samples. Bioanalysis.

[104] Zuur MA (2016). Dried blood spots can help decrease the burden on patients dually infected with multidrug-resistant tuberculosis and HIV. Eur Respir J.

[105] Kuhlin J (2018). Mass spectrometry for therapeutic drug monitoring of anti-tuberculosis drugs. Clin Mass Spectrom.

[106] Koster RA (2019). A volumetric absorptive microsampling LCMS/MS method for five immunosuppressants and their hematocrit effects. Bioanalysis.

[107] Veen J (2011). Harmonisation of TB control in the WHO European Region: the history of the Wolfheze workshops. Eur Respir J.

[108] World Health Organization (2021). WHO consolidated guidelines on tuberculosis. Module 2: Screening. Systematic screening for tuberculosis disease.

[109] Avaliani Z (2020). What is behind programmatic treatment outcome definitions for tuberculosis. Eur Respir J.

[110] Migliori GB (2022). Clinical standards for the diagnosis, treatment and prevention of TB infection. Int J Tuberc Lung Dis.

[111] Chesov D (2019). Failing treatment of multidrug-resistant tuberculosis: a matter of definition. Int J Tuberc Lung Dis.

[112] Migliori GB, Global Tuberculosis Network (GTN) (2019). Evolution of programmatic definitions used in tuberculosis prevention and care. Clin Infect Dis.

[113] World Health Organization (2013). Definitions and Reporting Framework for Tuberculosis – 2013 Revision.

[114] Uplekar M (2015). WHO’s new end TB strategy. Lancet.

[115] World Health Organization (2015). Implementing the End TB Strategy: the essentials.

[116] Akkerman OW (2018). Cross border, highly individualised treatment of a patient with challenging extensively drug-resistant tuberculosis. Eur Respir J.

[117] Guglielmetti L (2019). Multidisciplinary advisory teams to manage multidrug-resistant tuberculosis: the example of the french consilium. Int J Tuberc Lung Dis.

[118] Pontali E, Tadolini M, Migliori GB (2019). TB consilia and quality of tuberculosis management. Int J Tuberc Lung Dis.

[119] (2020). Erratum: Treatment of drug-resistant tuberculosis: an official ATS/CDC/ERS/IDSA clinical practice guideline. Am J Respir Crit Care Med.

